# TNF-*α* Antagonizes the Effect of Leptin on Insulin Secretion through FOXO1-Dependent Transcriptional Suppression of LepRb in INS-1 Cells

**DOI:** 10.1155/2022/9142798

**Published:** 2022-02-14

**Authors:** Yang Zhang, Weidong Jin, Dongyun Zhang, Changhai Lin, Haiyan He, Fengxin Xie, Lixia Gan, Weiling Fu, Lixiang Wu, Yongzhong Wu

**Affiliations:** ^1^Department of Laboratory Medicine, Chongqing University Cancer Hospital, Chongqing, China; ^2^Department of Laboratory Medicine, First Affiliated Hospital, Third Military Medical University (Army Medical University), Chongqing 400038, China; ^3^Department of Biochemistry and Molecular Biology, Third Military Medical University (Army Medical University), Chongqing 400038, China; ^4^Department of Radiotherapy, Chongqing University Cancer Hospital, Chongqing, China

## Abstract

Proinflammatory cytokines play a causal role in the development of hyperinsulinemia and T2MD. FOXO1, a transcription factor which is known to enhance proinflammation, was recently shown to be involved in obesity-induced *β* cell dysfunction. However, molecular mechanisms for the association remained elusive. In this study, we first found that both leptin (10 nM) and TNF-*α* (20 ng/ml) significantly inhibited glucose-stimulated insulin secretion (GSIS) of INS-1E cells. When in combination, the GSIS function of INS-1E cells was significantly increased compared with that of the leptin alone treatment, indicating that TNF-*α* attenuated the inhibiting effect of leptin on GSIS of INS-1E cells. Similarly, we found that TNF-*α* has the same inhibitory effect on leptin in regulating insulin synthesis and secretion, and the survival and apoptosis of insulin cells. Further studies showed that TNF-*α* blocks leptin pathway by reducing the expression of leptin receptor (LepRb, also called OBRb) and inhibiting the activation of STAT3, a key molecule involved in the leptin signaling pathway in INS-1E cells. Besides, the downregulated expression of phosphorylated FOXO1 was found to be involved in the possible mechanism of TNF-*α*. Overexpression of constitutively active FOXO1 markedly aggravated the LepRb reduction by TNF-*α* treatment of INS-1E cells, and the endogenous FOXO1 knockdown abolished the effect of TNF-*α* on INS-1E cells. Furthermore, we have proved that FOXO1 could directly bind to the promoter of LepRb as a negative transcription regulator. Taken together, the results of this study reveal that TNF-*α*-induced LepRb downregulated in pancreatic *β* cells and demonstrate that transcriptional reduction of FOXO1 might be the primary mechanism underlying TNF-*α* promoting INS-1E leptin resistance and *β* cell dysfunction. *Conclusions*. Our current studies based on INS-1E cells in vitro indicate that the inflammatory factor TNF-*α* plays an important role in the development of INS-1E leptin resistance and glucose metabolism disorders, probably through FOXO1-induced transcription reduction of LepRb promoter in pancreatic *β* cells, and FOXO1 may be a novel target for treating *β* cell dysfunction in obesity-induced hyperinsulinemia and T2DM.

## 1. Introduction

Obesity is defined as a body mass index (BMI)≧30 kg/m^2^ [1]. With this standard, the prevalence of obesity is increasing rapidly around the world and nearly a global epidemic [2, 3]. Accumulating findings indicate that obesity is one of the most important risk factors for health with cardiometabolic complications, including insulin resistance, metabolic syndrome components, type 2 diabetes (T2MD), and cardiovascular disease [4]. Insulin is a powerful anabolic hormone secreted from pancreatic *β* cells that acts on multiple tissues to stimulate the synthesis and storage of carbohydrates, lipids, and proteins. A recent study proved that obesity was also an independent predictor for fasting hyperinsulinemia [5]. It is reported that *β* cell dysfunction is a common result of hyperinsulinemia and insulin resistance during progression of metabolic syndrome to T2MD [6]. The role of *β* cell dysfunction in the pathogenesis of type 2 diabetes has gradually been revealed in recent years. However, the effects of obesity on *β* cell dysfunction remain unclear. Increasing findings report that obesity is strongly associated with the development of leptin resistance [7], which in turn plays a key role in the pathogenesis of T2MD, but the mechanism needs to be more elucidated.

The adipocyte factor leptin is derived from the Greek word “LEPTOS,” which means lean. It is mainly produced by adipocytes and in low levels by the gastric fundic epithelium, intestine, placenta, skeletal muscle, mammary epithelium, and brain [8]. Leptin levels in white adipose tissue and plasma are related to the energy store, such that leptin increases in obesity and decreases during fasting. Congenital leptin deficiency has been associated with hyperphagia, impaired thermogenesis, insulin resistance, hyperlipidemia, and central hypogonadism, which can be reversed by leptin treatment [9]. There are a large amount of leptin receptors expressed in pancreatic tissue [10, 11]. Studies have shown that significant hyperinsulinemia have occurred in leptin receptor knockout mice, confirming that leptin receptor pathway plays a crucial role in obesity-induced hyperinsulinemia [12]. Leptin resistance is defined by a reduced sensitivity or a failure in response of the brain to leptin, showing a decrease in the ability of leptin to reduce appetite or enhance energy expenditure, which causes an increased food intake and finally leads to overweight, obesity, cardiovascular diseases, and other metabolic disorders. Leptin resistance is a challenge for clinical treatment or drug discovery of T2MD [13].

Low-grade inflammation is accompanied by obesity and plays crucial roles in the progression of T2MD [14]. As a well-known monocyte-derived cytokine, tumor necrosis factor-*α* (TNF-*α*) plays an important role in mediating local and systemic inflammatory responses. TNF-*α* is also overexpressed in adipose tissue of obese animals and humans. It was reported that in animal models of obesity/insulin resistance, treatment with recombinant TNF-*α* receptor protein resulted in a 2- to 3-fold increase in insulin sensitivity [15, 16], and no insulin resistance was observed in obese mice that knocked out TNF-*α* or TNF-*α* receptors, indicating that adipose tissue-derived TNF-*α* is a major contributor of obesity-induced insulin resistance [17, 18]. In addition to inducing insulin resistance in insulin-responsive tissues, TNF-*α* also plays an important role in the pathogenesis of *β* cell dysfunction. Leptin, free fatty acids, adiponectin, TNF-*α*, and IL-6 are all produced and secreted by adipocytes and may directly influence aspects of *β* cell dysfunction, including insulin synthesis and secretion, and insulin cell survival and apoptosis. During the progression from normal weight to obesity and on to overt diabetes, TNF-*α* contribute to the occurrence and development of *β* cell dysfunction and T2MD. Nevertheless, the role of TNF-*α* in *β* cell dysfunction required to be further studied.

Forkhead box protein O1 (FOXO1), a member of forkhead box O (FOXO) family of transcription factors, regulates a wide range of molecular signals in tumor, liver, and brain and plays a vital role in cell apoptosis, inflammation, and tissue development [19]. Previous studies have reported that FOXO1 activation enhances the expression of proinflammatory cytokines, such as IL-1*β* and monocyte chemoattractant protein-1 during chronic inflammation [20]. It has been reported that FOXO1 is one of the terminal transcription factors of the insulin signaling pathway [21]. Impaired insulin signaling leads to decreased phosphorylation of FOXO1, which promotes its translocation from the cytoplasm to the nucleus for transcriptional activity. The role of FOXO1 in inflammatory and metabolic disorders has been widely investigated, while its function in obesity-induced *β* cell dysfunction during the development of T2MD remains to be elucidated.

In this study, we investigated the role and mechanism of TNF-*α* in *β* cell dysfunction and found that TNF-*α* inhibited the effects of leptin on GSIS, insulin synthesis and secretion, and *β* cell survival and apoptosis. Further studies showed that TNF-*α* achieves these effects by reducing the expression of leptin receptor LepRb. We present evidence that FOXO1, as a transcriptional regulation factor, is centrally involved in the regulation of TNF-*α* on LepRb expression. Our research is expected to provide new targets for the treatment of obesity-induced *β* cell dysfunction and T2DM.

## 2. Materials and Methods

### 2.1. Animals

Adult male SD rats (8~10 weeks) provided by the Experimental Animal Center of the Third Military Medical University were housed at a constant temperature (22 ± 1°C) and with a consistent light-dark cycle (light from 6 a.m. to 6 p.m.). Drinking water and commercial mouse food were available ad libitum. All animal procedures in this study were approved by the Ethics Committee of the Third Military Medical University (AMUWEC20210876).

### 2.2. Cell Line and Culture

INS-1E cells were grown in RPMI-1640 medium (Invitrogen, Carlsbad, CA, USA) supplemented with 10% FBS (Gibco, Gaithersburg, MD, USA) and antibiotics (100 U/ml penicillin and streptomycin). The HEK293 cells were cultivated in DMEM medium (Invitrogen) containing 10% FBS (Gibco). Cells were maintained at 37°C in a humidified 5% CO_2_ atmosphere.

### 2.3. Glucose-Stimulated Insulin Secretion Test

Insulin content was measured as described before [22]. Briefly, INS-1E cells were seeded on cell culture 24-well plates at a density of 5 × 10^5^ cells per well and cultured overnight. The cells were washed twice with PBS and incubated further with RPMI-1640 medium containing different concentrations of either TNF-*α* (0, 2, and 20 ng/ml; R&D Systems, Oxon, UK), or leptin (0, 0.5, and 10 nM; R&D Systems, Oxon, UK), or combination of TNF-*α* and leptin. After 48 h of incubation, the cells were washed twice with Krebs-Ringer bicarbonate buffer (KRBB, 129 mM NaCl, 4.8 mM KCl, 1.2 mM MgSO_4_, 1.2 mM KH_2_PO_4_, 2.5 mM CaCl_2_, 5 mM NaHCO_3_, 0.1% BSA, and 10 mM HEPES [pH 7.4]) and starved for 2 hours in KRBB, and then incubated for 1 h in KRB buffer containing 3.3 mM or 16.7 mM glucose. The supernatants were collected and centrifuged (1000 rpm, 5 min) to measure insulin content using rat insulin ELISA kits (Linco Research, Inc., St. Charles, MO, USA) according to the kit's instructions.

### 2.4. Isolation and Cultivation of Primary Islets of Rats

Islets were isolated by collagenase digestion and Ficoll purification from the pancreas of adult male SD rats according to the previous report [23]. Briefly, rats were anesthetized by 2% pentobarbital sodium injection. The pancreas and common bile duct were fully exposed. Then, the entrance of common bile duct to duodenum was clamped; the duct was cannulated with a polyethylene catheter. 10 ml cold Hanks buffer containing a small amount of collagenase was gently injected into the duct. The inflated pancreas was removed and digested with collagenase at 38°C for 30 minutes and then followed by stopping the digestion with cold Hanks solution. The solution was centrifuged at 1500*g* for 2 minutes at 4°C, the supernatant was removed, and the islets were purified by discontinuous Ficoll density gradient centrifugation. Purified islets were washed with cold RPMI-1640 medium and centrifuged at 4°C, 1,000*g* × 4 min for 2 times. Then, the purified islets were transferred into a 48-well cell culture plate with a destiny of 10 islets/well and cultured overnight in RPMI-1640 medium (3.3 mM glucose + 10%FBS) at 37°C with 5% CO_2_. After overnight incubation, groups of each containing ten size-matched islets were glucose-starved by incubation at 37°C for 1 h in HEPES buffer (pH 7.4) containing (in mM) 125 NaCl, 5.9 KCl, 1.2 MgCl_2_, and 1.28 CaCl_2_, supplemented with 3 mM glucose and 1 mg/ml BSA. Then, islets were switched to a solution of the same buffer containing either 3.3 mM (control) or 16.7 mM (stimulated) glucose for GSIS test. The experimental grouping and drug treatment were consistent with INS-1E cells.

### 2.5. RNA Isolation and Quantitative Real-Time PCR

Total RNA from INS-1E cells were isolated using TRIzol reagent (Invitrogen, Carlsbad, CA, USA) according to the instructions of the manufacturer. RNAs were transcribed to cDNA at 42°C for 1 hour in a 25 *μ*l cocktail containing 5× reverse transcriptase (RT) buffer, 10 mM dNTPs (200 units), Moloney murine leukemia virus RT (Promega, Madison, WI, USA), and 100 pm oligo-dT primer. After the termination of cDNA synthesis, the mRNA levels of target genes were determined by quantitative RT-PCR as described previously [24]. The relative amounts of the mRNA levels of the target genes were normalized to *β*-actin, and the relative difference in mRNA levels was calculated by the 2^-*ΔΔ*Ct^ method. The sequences of primers and reaction conditions used are listed in Table S1 and Table S2.

### 2.6. Western Blotting

INS-1E cells treated with leptin (10 nM) or/and TNF-*α* (20 ng/ml) were collected, and total protein extracts were prepared as previously described [25]. Briefly, 50 *μ*l of total protein sample was subjected to 12% SDS-PAGE and transferred overnight at 4°C onto PVDF membranes (Millipore, Bedford, MA, USA). The membranes were blocked with blocking buffer (20 mM Tris, 150 mM NaCl, pH 7.5, and 5% nonfat dry milk) at room temperature for 1 hour and then incubated with primary antibodies against p-STAT3 (1 : 500, Santa Cruz Biotechnology, CA, USA, sc-8059), STAT3 (1 : 1000, Santa Cruz Biotechnology, CA, USA, sc-8019), LepRb (1 : 400, Santa Cruz Biotechnology, CA, USA, sc-8325), p-FOXO1[Ser^256^](1 : 1000, Cell Signaling Technology, MA, USA, 9461S), FOXO1 (1 : 1000, Cell Signaling Technology, MA, USA, 9454S), SOCS3 (1 : 500, Santa Cruz Biotechnology, CA, USA, sc-73045), and *β*-actin (1 : 1000, Santa Cruz Biotechnology, CA, USA, sc-47778). The antigen-antibody complex was then detected by incubating the membranes for 1 hour in a buffer containing a 1 : 5000 dilution of horseradish peroxidase-conjugated goat anti-rabbit IgG secondary antibodies (Millipore, Billerica, MA, USA).

### 2.7. MTT and Flow Cytometry Apoptosis Assay

Cell proliferation of INS-1E cells was determined by means of a colorimetric MTT-assay as previously reported [26]. Briefly, INS-1E cells were serum-deprived overnight and exposed to leptin or/and TNF-*α* for 48 h. Then, the cells were incubated with 20 *μ*l 3-(4,5-dimethylthiazol-2-yl)-2,5-diphenyl-tetrazolium bromide (MTT; Sigma-Aldrich, St. Louis, MO, USA) at 37°C for 3 h. After removal of MTT, 150 *μ*l DMSO was added to the cells and the absorbance was measured at 492 nm with a microplate reader. Apoptosis assay was performed with Annexin V-FITC apoptosis assay kit (Bender MedSystems) in accordance with the manufacturer's directions.

### 2.8. Plasmid Construction

**Figure 1:**
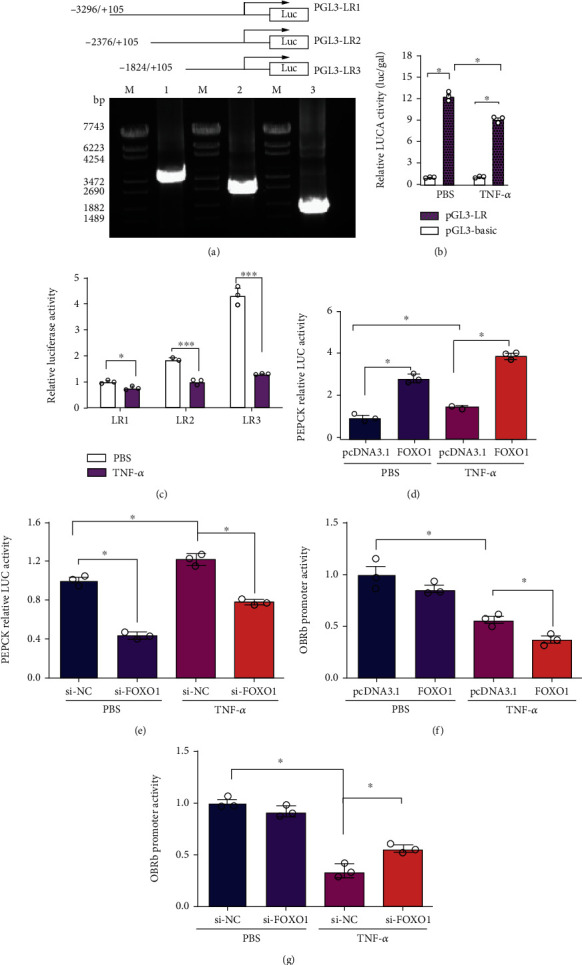
TNF-*α* transcriptionally reduced LepRb expression in a FOXO1-dependent way. (a, b) A schematic depiction of different LepRb promoter regions cloned into the pGL3-basic plasmid. The constructs were designated as LR1, LR2, and LR3. (c) Effects of TNF-*α* on promoter activity of different promoter fragment in HEK293 cells treated with TNF-*α* (20 ng/ml) for 24 h. Results are expressed as the relative luciferase activity normalized by the activity from the pGL3-LR1 transfected group without TNF-*α* treatment (the first bar), which are arbitrarily defined as 1. (d) TNF-*α* has regulatory effect on the PEPCK promoter activity, a known target gene of FOXO1. A 593 bp (-523/+70) DNA fragment harboring the human PEPCK promoter was cloned into pGL3-basic vector and used for luciferase assays, after cotransfection with either FOXO1-TSS or the control plasmid pcDNA3.1 followed by TNF-*α* or PBS treatment for 24 h. (e) The PEPCK promoter construct was cotransfected with FOXO1 siRNA (siFOXO1) or siNC into HEK293 cells, and luciferase activities were assayed 48 h after transfection with or without TNF-*α* treatment for 24 h. (f, g) LR3 (-1824/+105) DNA fragment was cotransfected with either FOXO1-TSS plasmid or pcDNA3.1 (control), and FOXO1 siRNA (siFOXO1) or control siRNA (siNC). Cells were treated 6 h after transfection with TNF-*α* (20 ng/ml) for an additional 24 h and then harvested for luciferase assays. Data are presented as the means ± SEM of *n* = 3; ^∗^*P* < 0.05 vs. controls.

Human FOXO1-AAA plasmid, a constitutive active form construct with the three conserved Akt/PKB sites (Thr24, Ser 256, and Ser319) within FOXO1 specifically mutated into alanine, was kindly provided by Dr. Terry Unterman (University of Illinois at Chicago). Both FOXO1-TSS plasmid and pcDNA3.1 were preserved in our laboratory. The DNA fragments for the three human LepRb promoter fusion reporter constructs shown in Figure 1(a) were generated from human genomic DNA by PCR amplification using KOD Plus (Toyobo). The primers used for the DNA fragments are listed in Additional file 1: Table S3. LepRb gene sequence blast was carried using NCBI database, and a region encoding human LepRb gene (NC_000001.9, chr1:65655540-65659010) transcription initiation site upstream -3296 bp to downstream +105 bp containing promoter and region of proximal regulatory sequences were found.

### 2.9. Cell Transfection and Luciferase Reporter Assays

Amplified DNA fragments were inserted into the pGL3-Basic vector. Transient transfections were conducted using Lipofectamine 2000 according to the protocol as previously reported [27]. Briefly, INS-1E cells were plated 24 h before transfection on a 24-well culture dish at a density of 5 × 10^5^/well and then transfected with 100 *μ*l of LepRb reporter gene plasmid or equal molar amounts of its truncated promoter reporter constructs. After transfection for 6 h, the medium was removed and replaced with complete growth medium containing 20 ng/ml TNF-*α* or PBS as control. After 36 hours of incubation, cells were washed twice and lysed with specific reporter lysis buffer. Then, the luciferase activities of the cell lysate were evaluated according to the manufacturer's instructions (Promega Corp., Madison, WI), and the total protein concentration in each well was measured as an internal control.

### 2.10. Chromatin Immunoprecipitation (ChIP) Assay

MAGnify™ System (Thermo Fisher Scientific) was used for ChIP assay [28]. Briefly, a total of 5 × 10^6^ INS-1E cells were seeded in 6-well dishes and normally cultured until 80% confluence and then harvested in PBS after cross-linking with 1% formaldehyde at room temperature for 10 min. Cells were then collected and transferred to a centrifuge tube. After centrifugation, the cells were incubated with SDS lysis buffer supplemented with proteinase inhibitor for 1 h at 4°C. The mixture was sonicated on ice to obtain 200 to 500 base pair DNA fragments. The ChIP assay was performed according to the manufacturer's protocol (Millipore Corp). The input group accounted for 1% of the total DNA, while the IgG and FOXO1 groups were added with their ChIP grade antibodies. The immunoprecipitates were analyzed by PCR for detecting the protein binding sites of the DNA samples. The primers were designed as follows: LR1 promoter forward: GCACGCGTGAATGGATTTGATGCCCTGT, LR1 promoter reverse: GCCTCGAGTTTAACGCCCGCCATGTCT; LR2 promoter forward: GCACGCGTCAGGGCTTGTCTGATACGCAGG, LR2 promoter reverse: GCCTCGAGTTTAACGCCCGCCATGTCT; and LR3 promoter forward: GCACGCGTGTGCTTTTCTAGTACCGTGTAC, LR3 promoter reverse: GCCTCGAGTTTAACGCCCGCCATGTCT. The calculation of the ChIP signal is %input = 1% × 2^(CTinput − CTsample)^.

### 2.11. Site-Directed Reporter Gene Mutation

FOXO1-specific binding sites in the human LepRb promoter region LR3 were mutated using MutanBEST Kit (Takara Bio, Inc., Shiga, Japan) according to the manufacturer's protocol. Briefly, the required primers were designed as follows: for LR3-mut1, forward: GCATCAGCAGTGAGTCAGGTA, reverse: TCTCCTCATAGACGGATGGCA; for LR3-mut2, forward: GACATTTCTTGAAACAGTAACATGC, reverse: GTTTCATGATCCTTGCTCAGTGG; for LR3-mut3, forward: CCCGCTAGTTTCAGTAAAGCG, reverse: CCCCTTAGGAAACACTCCTCA; and for LR3-mut4, forward: GCGGCCTCTGCGAGC, reverse: GGCGCCTGCTGCTCC.

### 2.12. DNA Pull-Down Assay

The DNA pull-down was performed with a DNA pull-down test kit (Thermo Fisher Scientific) according to the manual [29]. Briefly, LR3 and its four mutational sequences were amplified by PCR and tagged with biotin. The biotin-labeled promoters were bound with streptavidin magnetic beads (Dynabeads™ M-280 streptavidin; Thermo Fisher Scientific, Inc.) at 4°C for 4 h. The nonbiotinylated promoter was used as the negative control. All proteins extracted from INS-1E cells in the input group were used as the positive control. The bound promoter was incubated with 1 mg protein extracted from INS-1E cells with gentle agitation at 4°C overnight. The bound beads-promoter protein complexes were eluted and subsequently subjected to Western blot assay for FOXO1 detection.

### 2.13. FOXO1 Knockdown by RNA Interference

Human FOXO1-specific siRNA (si-FOXO1): 5-AAGCCCTGGCTCTCACAGC-AA-3 and the scrambled control RNA (siNC): 5-UUCUCCGAACGUGUCACGUT-T-3 were designed to knock down the expression of endogenous FOXO1 genes according to our previous report [27]. INS-1E cells were transfected using Lipofectamine 2000 in accordance with the manufacturer's instructions. TNF-*α* (20 ng/ml) was added for an additional 24 h after 48 h of transfection with 20 nmol/ml of siRNA.

### 2.14. Statistics

Statistical analysis was performed using software SPSS V18 (SPSS Inc., Chicago, IL, USA). All measurement data were presented as the mean ± SEM. The significance of each group was analyzed using one-way analysis of variance (ANOVA) followed by Tukey's multiple comparison post hoc test. *P* < 0.05 was considered significant.

## 3. Results

### 3.1. TNF-*α* Inhibited the Effect of Leptin on Glucose-Stimulated Insulin Secretion (GSIS) in INS-1E Cells and Pancreatic Islets

**Figure 2:**
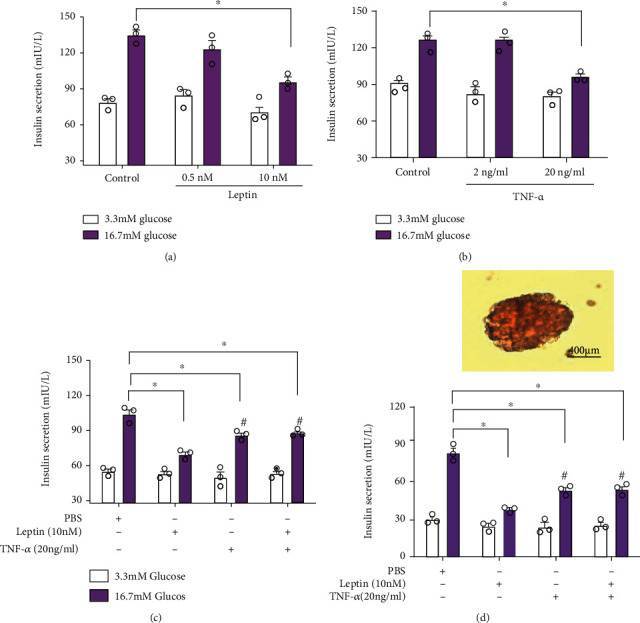
TNF-*α* attenuated the inhibitory effects of leptin on GSIS in *β* cells. INS-1E cells or islets were treated with different dose of leptin and TNF-*α* and then harvested for the determination of GSIS. (a) Intracellular insulin content of INS-1E cells treated with different concentrations of leptin. (b) Intracellular insulin content of INS-1E cells treated with different concentrations of TNF-*α*. (c) Intracellular insulin content of INS-1E cells treated with leptin (10 nM) and TNF-*α* (20 ng/ml) alone or in combination. (d) Intracellular insulin content of islet cells treated with leptin (10 nM) and TNF-*α* (20 ng/ml) alone or in combination, upper: micrograph of isolated pancreatic islets of rats. Data are presented as the means ± SEM of *n* = 3; ^∗^*P* < 0.05 as compared to the vehicle group and ^#^*P* < 0.05 as compared to the leptin group.

To identify the molecular linking between TNF-*α* and leptin under the state of obesity, we firstly investigated the effects of leptin and TNF-*α* on the GSIS function in INS-1E cells. Results showed that leptin (10 nM) and TNF-*α* (20 ng/ml) alone could significantly reduce the GSIS function of INS-1E cells (Figures 2(a) and 2(b)). However, when INS-1E cells were treated with leptin (10 nM) and TNF-*α* (20 ng/ml) in combination, the GSIS function of INS-1E cells was significantly increased compared with the leptin alone treatment group (Figure 2(c)). We further determined this effect of TNF-*α* in rat pancreatic islets, and similar results were found (Figure 2(d)). These results demonstrated that TNF-*α* attenuates the inhibitory effect of leptin on GSIS of *β* cells.

### 3.2. TNF-*α* Antagonized the Regulation of Leptin on Insulin Transcription and Proapoptosis in INS-1E Cells

**Figure 3:**
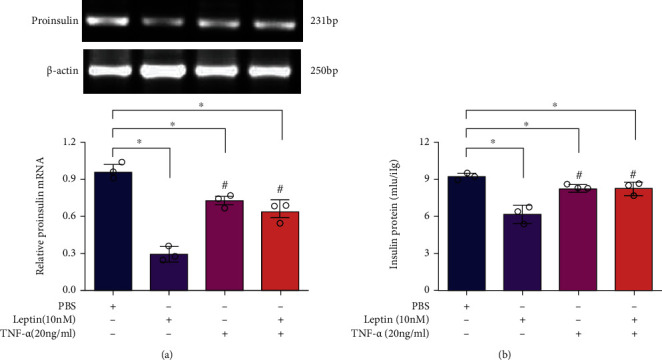
TNF-*α* reduced the effects of leptin on insulin expression in INS-1E cells. Cells were treated with leptin (10 nM) and TNF-*α* (20 ng/ml) alone or in combination, and then, proinsulin mRNA and secreted insulin levels were determined. (a) Relative proinsulin mRNA levels in INS-1E cells determined by PCR; upper: bands of proinsulin expression determined by RT-PCR and gel electrophoresis, lower: quantification of proinsulin mRNA by real-time PCR. (b) Secreted insulin protein in INS-1E cells determined by ELISA kit. Data are presented as the means ± SEM of *n* = 3; ^∗^*P* < 0.05 as compared to the vehicle group and ^#^*P* < 0.05 as compared to the leptin group.

To further explore the role of TNF-*α* in inhibiting the effect of leptin on GSIS at the gene level, we examined the proinsulin mRNA levels and insulin protein levels of INS-1E cells treated with leptin (10 nM) and TNF-*α* (20 ng/ml). RT-PCR results showed that the expression of proinsulin mRNA was reduced by leptin, and TNF-*α* significantly reversed the inhibitory of leptin on proinsulin mRNA (Figure 3(a)). Similarly, ELISA results showed that leptin notably inhibited the level of insulin protein, and this effect was significantly reversed by TNF-*α* (Figure 3(b)). These data indicated that TNF-*α* antagonized the action of leptin on GSIS by regulating insulin gene expression.

**Figure 4:**
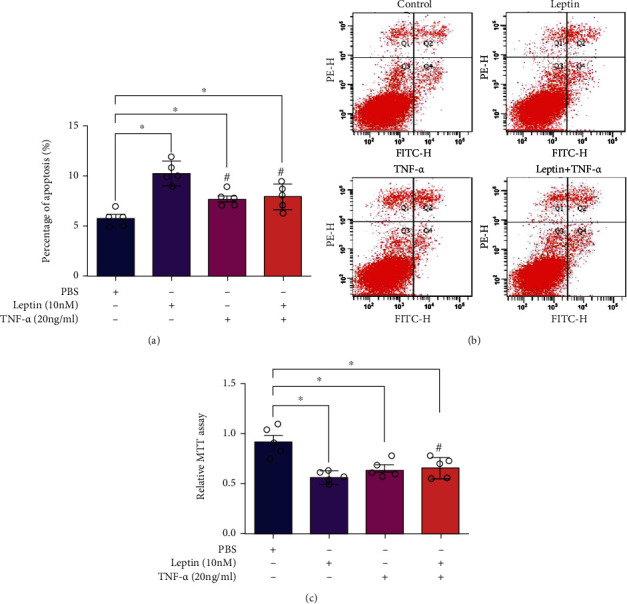
TNF-*α* antagonized the effects of leptin on the proliferation of INS-1E cells. INS-1E cells in 24-well plates at a density of 5 × 10^5^ per well were treated with leptin (10 nM) and TNF-*α* (20 ng/ml) alone or in combination. After a 48-hour incubation, the proliferations of cells were determined. (a) The statistic percentage of apoptosis of treated INS-1E cells. (b) The apoptosis of INS-1E cells detected by flow cytometry. (c) The survival rate of INS-1E cells determined by MTT assay. Data are presented as the means ± SEM of *n* = 5; ^∗^*P* < 0.05 as compared to the vehicle group and ^#^*P* < 0.05 as compared to the leptin group.

The regulation of *β* cell mass is essential for the compensatory response of the endocrine pancreas to situations of increased insulin demand such as obesity. *β* cell mass in subjects with obesity is assumed to increase, since plasma insulin levels in obese subjects increase to compensate for insulin resistance, a process known as hyperinsulinemia [30]. In order to study the role of TNF-*α* and leptin in the regulation of *β* cell mass, INS-1E cells were treated with leptin (10 nM) and (20 ng/ml) alone or in combination, and then, the percentage of apoptosis and cell viability were measured. Flow cytometry results showed that both leptin (10 nM) and TNF-*α* (20 ng/ml) significantly accelerated the apoptosis of INS-1E cells. However, when INS-1E cells were treated with leptin (10 nM) and TNF-*α* (20 ng/ml) in combination, the percentage of apoptosis was relatively decreased compared with those treated with leptin (10 nM) but not with TNF-*α* (20 ng/ml) (Figures 4(a) and 4(b)). Meanwhile, the evaluation of cell viability by MTT assay showed that TNF-*α* attenuated the effect of leptin on the viability of INS-1E cells (Figure 4(c)). The results of MTT were perfectly according with those of apoptosis, and both consistently confirmed that TNF-*α* antagonized the regulation effect of leptin on *β* cell mass.

### 3.3. TNF-*α* Downregulated LepRb Expression and the following JAK2/STAT3 Pathway in INS-1E Cells

**Figure 5:**
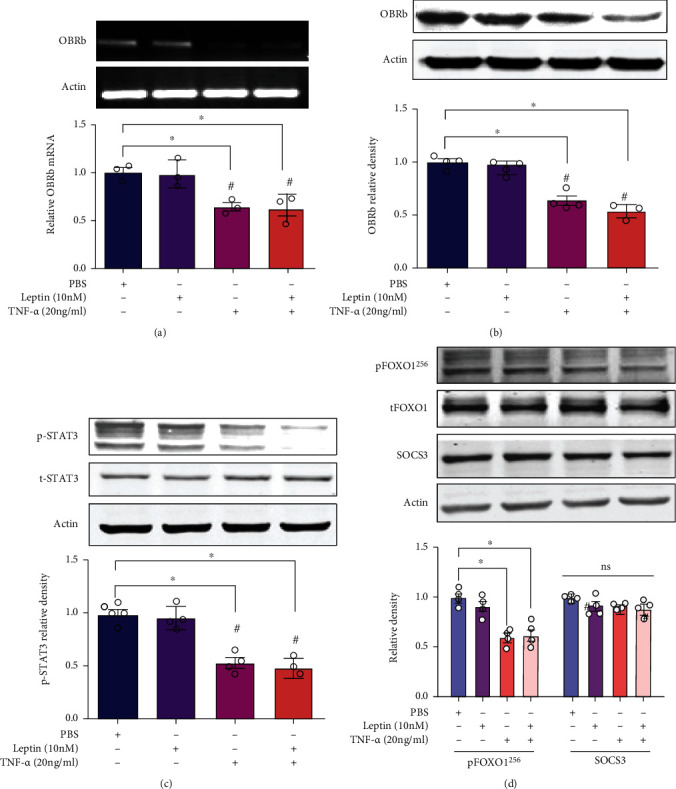
TNF-*α* reduced the effect of leptin via the FOXO1 pathway. INS-1E cells in six-well plates were treated with leptin (10 nM) and TNF-*α* (20 ng/ml) alone or in combination, and cells were harvested for mRNA and protein analyses 48 h after incubation. (a) Relative LepRb mRNA levels in INS-1E cells determined by RT-PCR. Data are presented as the means ± SEM of *n* = 3. (b) Relative LepRb protein in INS-1E cells determined by Western blotting. Data are presented as the means ± SEM of *n* = 4. (c) Relative phosphorylated STAT3 levels in INS-1E cells determined by Western blotting. Data are presented as the means ± SEM of *n* = 4. (d) Relative pFOXO1 and SOCS3 levels in INS-1E cells determined by Western blotting. Actin was as an internal control. Data are presented as the means ± SEM of *n* = 4; ^∗^*P* < 0.05 as compared to the vehicle group and ^#^*P* < 0.05 as compared to the leptin group.

Previous studies demonstrated that leptin exerts a range of biological functions through the long form of leptin receptor (LepRb), which play a key role in the leptin signaling pathway [31, 32]. To determine the role of TNF-*α* in the leptin pathway, we further examined the regulation of TNF-*α* on LepRb expression. By using RT-PCR and Western blotting, we found that TNF-*α* (10 nM) significantly inhibited the expression level of LepRb (Figures 5(a) and 5(b)), despite with or without leptin. The data illustrated that TNF-*α* abolished the regulatory effect of leptin on insulin secretion of *β* cells via inhibiting the expression of LepRb.

### 3.4. TNF-*α* Reduced the Effect of Leptin on *β* Cells via the FOXO1 Pathway

The Janus kinase-2 (JAK2) and signal transducer and activator of transcription (STAT3) pathway were regarded as a major leptin signaling pathway. To investigate the effects of TNF-*α* stimulus on the signaling cascade of the leptin receptor, we studied the activation of STAT3 in INS-1E cells treated with leptin and TNF-*α*. Intriguingly, the level of p-STAT3 was not further increased by leptin (10 nM) compared with the PBS group (Figure 5(c)). This is probably because of the effect of leptin on p-STAT3 activation was concealed by high glucose containing in INS-1E cell medium, because previous studies reported that high glucose could also induce the activation of p-STAT3 in different types of cells [33, 34]. However, we found that TNF-*α* (20 ng/ml) successfully inhibited the activation of STAT3 (Figure 5(c)), indicating that TNF-*α* reduced the effect of the leptin signaling pathway. To define the mechanism and role of TNF-*α* in inhibiting the regulation effect of leptin in *β* cell functions, we further studied the phosphorylation level of FOXO1 in INS-1E cells treated with TNF-*α*. Our results showed that TNF-*α* (20 ng/ml) significantly decreased the expression level of p-FOXO1 (Figure 5(d)), suggesting that TNF-*α* reduced the leptin pathway probably by inhibiting the phosphorylation of FOXO1.

Number of studies show that the activated STAT3 translocate into the nucleus where it mediates the expression of target genes including suppressor of cytokine signaling 3 (SOCS3), which is an inhibitor of OBR signaling [35–37]. To explore whether TNF-*α* inhibit the effect of leptin by directly mediating SOCS3, we next examined the expressing level changes of SOCS3 in INS-1E cells treated with TNF-*α*. However, no significant effect on the expression of SOCS3 was observed (Figure 5(d)), indicating that SOCS3 was not involved in the regulation of TNF-*α* on leptin signaling.

### 3.5. FOXO1 Overexpression Aggravated the Effect of TNF-*α* on LepRb Suppression in INS-1E Cells

**Figure 6:**
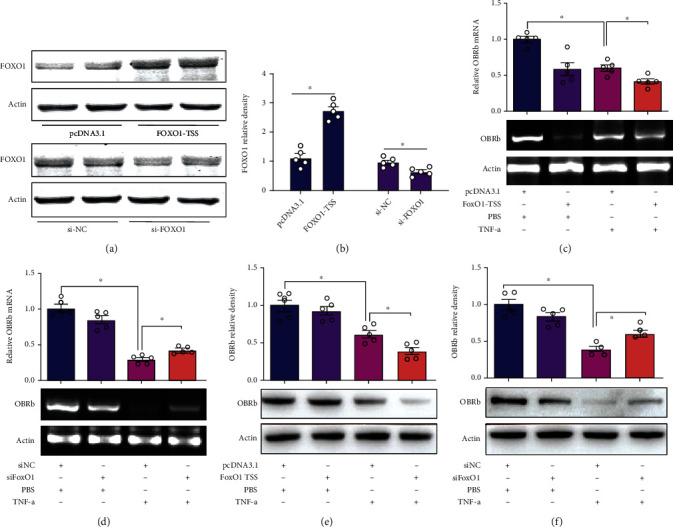
Ectopic overexpression of FOXO1 enhanced the inhibitory effect of TNF-*α* on LepRb expression, and FOXO1 silencing abolished the effect of TNF-*α* on LepRb gene. INS-1E cells were transfected with a plasmid expressing constitutively active FOXO1 or a control vector pcDNA3.1 or were transfected with siFOXO1 or scrambled siRNA (siNC) as a control. Cells were harvested 48 h after transfection, and the endogenous FOXO1 expressions were measured by Western blotting. (a) FOXO1 protein levels determined by Western blotting. (b) The statistic results of endogenous FOXO1 expression. Cells were treated with leptin (10 nM) and TNF-*α* (20 ng/ml) alone or in combination for 24 h after transfection and then harvested for RT-PCR and Western blotting analysis of LepRb. Data are presented as the means ± SEM of *n* = 8. (c) Relative LepRb mRNA levels in INS-1E cells overexpressing FOXO1. Data are presented as the means ± SEM of *n* = 5. (d) Effects of FOXO1 silencing on TNF-*α* reduced LepRb mRNA levels in INS-1E cells. Data are presented as the means ± SEM of *n* = 5. (e) Relative LepRb protein levels in HEK293 cells determined by Western blotting. Data are presented as the means ± SEM of *n* = 5. (f) Effects of FOXO1 silencing on LepRb protein levels in INS-1E cells determined by Western blotting. Data are presented as the means ± SEM of *n* = 5; ^∗^*P* < 0.05 vs. controls.

In order to identify the mechanism role of FOXO1 in LepRb gene expression, INS-1E cells were transfected with a human FOXO1-TSS plasmid expressing active FOXO1 constitutively or a pcDNA3.1 plasmid as the control vector. After 48 hours of transfection, the endogenous FOXO1 expression of INS-1E cells was determined by Western blotting. Data showed that ectopic gene transfer significantly increased the expression of FOXO1 in INS-1E cells (Figures 6(a) and 6(b)). As an initial step to confirm the effect of FOXO1 on LepRb gene regulation, we measured both LepRb mRNA and protein levels in INS-1E cells treated with or without TNF-*α*. The results showed that FOXO1 overexpression significantly decreased LepRb mRNA levels (Figure 6(c)) and protein levels (Figure 6(e)) in TNF-*α*-treated INS-1E cells.

### 3.6. FOXO1 Knockdown Abolished the Effects of TNF-*α* on LepRb Suppression in INS-1E Cells

To confirm the role of FOXO1 in TNF-*α*-mediated LepRb gene expression, small interfering RNA (siRNA) specifically targeting human FOXO1 (siFOXO1) was transfected into INS-1E cells to silence endogenous FOXO1 expression in INS-1E cells (Figure 6(a)). The results testified that the FOXO1 expression was reduced in INS-1E cells transfected with siFOXO1, which compared with scrambled siRNA (siNC) (Figure 6(b)). After 48 h of transfection with siRNA, INS-1E cells were treated with TNF-*α* (20 ng/ml) for an additional 24 h; the result showed that FOXO1 silencing significantly attenuated the inhibitory effect of TNF-*α* on LepRb mRNA levels (Figure 6(d)) and protein levels (Figure 6(f)). These data suggested that the inhibitory effect of TNF-*α* on the expression of LepRb is FOXO1-dependent.

### 3.7. TNF-*α* Transcriptionally Reduced LepRb Expression in a FOXO1-Dependent Way

In order to explore how FOXO1 enhances the inhibition of LepRb expression by TNF-*α* at the molecular level, three reporter constructs named LR1 (-3296/+105), LR2 (-2376/+105), and LR3 (-1824/+105) harboring different LepRb promoter regions were generated (Figure 1(a)). Reporter assays reveal that TNF-*α* has a significant inhibitory effect on the transcriptional activity of LR (LR1, LR2, and LR3) (Figure 1(b)), and with the truncation of the promoter fragment, the inhibitory effect of TNF-*α* on the transcriptional activity of the LepRb promoter reporter gene vector is significantly enhanced (Figure 1(c)).

Then, LepRb promoter activity was examined in HEK293 cells overexpressing constitutively active FOXO1 or treated with FOXO1 siRNA. The promoter of human phosphoenolpyruvate carboxykinase (Pepck) gene, a known target of FOXO1, was used as a positive control for the transcription activity of FOXO1 in this experiment. As expected, FOXO1 overexpression increased (Figure 1(d)), and silencing of endogenous FOXO1 decreased (Figure 1(e)) Pepck promoter activity significantly in HEK293 cells regardless of TNF-*α* treatment, confirming the FOXO1 transcriptional activity on target gene expression. Intriguingly, opposite effects of FOXO1 on LepRb promoter activity were observed when HEK293 cells were treated with TNF-*α* using a LR3 (-1824/+105) DNA fragment (Figures 1(f) and 1(g)). These results indicate that TNF-*α* has a negative regulatory effect on the LepRb promoter activity, depending on the transcriptional suppression of FOXO1.

### 3.8. FOXO1 Directly Binds to LepRb Promoter to Transcriptionally Reduce LepRb

**Figure 7:**
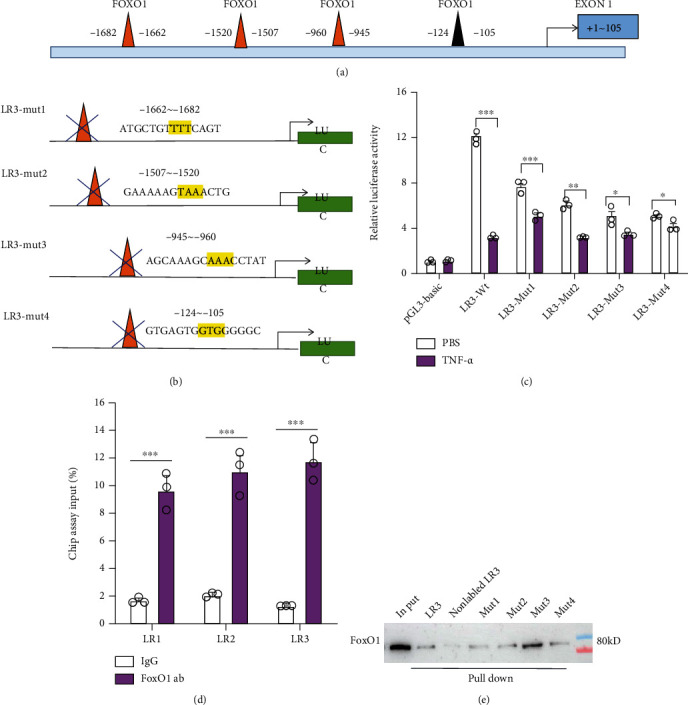
FOXO1 may transcriptionally contribute to aggravate the reduction of LepRb in HEK293 cell. (a) Prediction of FOXO1 binding sites at the promoter region (-1824/+105) of LepRb and design of four mutated fragments for LepRb promoter binding sites analysis (b). (c) The transcriptional activity of the indicated fragments for LepRb promoter was analyzed by luciferase reporter assay in HEK293 cells. (d) FOXO1 directly binds to the promoter of LepRb and ChIP-PCR which was employed to determine the interaction between FOXO1 protein and LepRb promoter. (e) DNA pull-down assay was performed to determine the interaction between indicated DNA fragments and FOXO1 protein. Data are presented as the means ± SEM of *n* = 3; ^∗^*P* < 0.05, ^∗∗^*P* < 0.01, and ^∗∗∗^*P* < 0.001 vs. controls.

We next investigated the mechanism underlying FOXO1 regulating LepRb. As FOXO1 primarily functions as a transcription factor, we speculated that FOXO1 could directly regulate LepRb at the transcriptional level. To confirm the binding site of FOXO1 on LepRb, we predicted the potential binding sites of FOXO1 on the LR3 promoter according to JASPAR (https://jaspar.genereg.net/) database and found that there are four potential FOXO1 binding sites in LR3, named region 1 (-1682 bp to -1662 bp), region 2 (-1520 bp to -1507 bp), region 3 (-960 bp to -945 bp), and region 4 (-124 bp to -105 bp) (Figure 7(a), Figure S1). We next prepared four mutated LR3 promoters: LR3-mut1, LR3-mt2, LR3-mt3, and LR3-mt4, respectively (Figure 7(b)). Then, the luciferase reporter assay was employed to examine the TNF-*α* response of these LepRb promoters. We found that all LR3-mut1, LR3-mut2, LR3-mut3, and LR3-mut4 fragments of LepRb promoter showed significantly reduced luciferase activity after treating with TNF-*α* (Figure 7(c)), indicating that all regions 1 to 4 are responsible for the effect of TNF-*α* on LepRb promoter.

To verify the direct binding between FOXO1 and LepRb promoter, we performed ChIP-PCR assay. Results showed that all of the three LepRb promoter regions were detected by qRT-PCR from the DNA fragments eluted from FOXO1-bound beads, which indicated the direct binding between FOXO1 and LepRb promoter (Figure 7(d)). Next, we performed DNA pull-down assay with WT and mutated LR3 promoters. We found that WT and Mut1-4 mutated LR3 promoters showed a significant binding with FOXO1 (Figure 7(e)), confirming that FOXO1 directly binds to LR3 promoter and regions 1 to 4 were responsible for this process.

## 4. Discussion

The current study provides evidence that TNF-*α* disturbed the regulation of leptin on *β* cell functions including GSIS, insulin synthesis and secretion, and *β* cell survival and apoptosis. Further studies confirmed that TNF-*α* could inhibit the expression of LepRb located on the surface of *β* cells. Besides, we explored the potential mechanism effect of FOXO1 on TNF-*α*-induced *β* cell dysfunction and proved that FOXO1 plays a crucial role in the regulation of TNF-*α* in the expression of LepRb by directly binding to the promoter of LepRb as a negative regulatory transcription factor. This study thus provides a novel mechanism that FOXO1 involves in proinflammatory cytokine-induced *β* cell dysfunction and would be a new target for the treatment of T2DM.

T2DM is characterized by a progressive decrease of insulin sensitivity and pancreatic *β* cell dysfunction. Both obesity and T2DM have been considered as a chronic low-grade inflammatory [38]. It is well established that proinflammatory cytokines disrupt insulin signaling at the insulin receptor and insulin receptor substrate (IRS) levels through multiple signaling pathways. Among the proinflammatory cytokines, TNF-*α* is well characterized to induce insulin resistance in cultured adipocytes and in mouse [21]. TNF-*α* may also have a role in impaired *β* cell function. In vitro studies have shown that TNF-*α* can reduce glucose-induced insulin release from pancreatic *β* cells by activating nuclear factor kappaB (NF-*κ*B) and local production of nitrous oxide [39]. Previous studies showed that decreased TNF-*α* levels after transient insulin therapy correlate with improved *β* cell function in type 2 diabetics; however, the mechanism effect of TNF-*α* on *β* cell function remains to be established.

Numerous studies have reported that obesity, insulin resistance, glucocorticoids, estrogens, and chronic inflammation are closely related to the high levels of leptin [38, 40]. In pancreas-specific leptin receptor knockout mice, GSIS is impaired by high-fat feeding, and the ability to compensate for islet hypertrophy is reduced, resulting in impaired glucose tolerance [41]. This confirms the importance of the leptin signaling pathway in maintaining the homeostasis of glucose metabolism and reveals that the loss of leptin receptor may be a fatal factor leading to *β* cell dysfunction in the course of T2DM. It has been reported that the transcription of proinsulin mRNA is inhibited by TNF-*α* and leptin [42]. In the present study, we found that both TNF-*α* (20 ng/ml) and leptin (10 nM) significantly reduced the GSIS function of *β* cells and inhibited the expression of insulin in gene level, which is consistent with previous reports. Intriguingly, when combined with TNF-*α*, the inhibitory effect of leptin on the transcription of proinsulin gene and the synthesis of insulin was significantly attenuated. Previous studies have reported that high concentration of leptin induces activation of NF-*κ*B through the MAPK/JNKs pathway, and TNF-*α* cause cell-selective destruction by promoting NO production and inducing apoptosis [43, 44].

Additionally, the regulation of *β* cell mass is essential for the compensatory response of the endocrine pancreas to situations of increased insulin demand such as obesity. Previous study reported that the mass of *β* cell increased during insulin resistance [45]. Other research reports that *β* cell mass in subjects with obesity is assumed to increase, since plasma insulin levels in obese subjects increase to compensate for insulin resistance, a process known as hyperinsulinemia [30]. Our current study has confirmed that TNF-*α* promote *β* cell mass, thus leading to overabundance of cells. These results are consistent with some previous reports. Therefore, we hypothesize that TNF-*α* is a potential inducer to deteriorate hyperinsulinemia and subsequent T2DM by promoting *β* cell mass and insulin secretion.

JAK/STAT is a vital pathway mediating the leptin signaling. JAK2, a member of the JAK family of protein tyrosine kinases, is the major subtype that recognizes and binds to the membrane proximal specific region of LepRb, which is considered the primary receptor for leptin [46]. Studies in obese rodents suggested that leptin resistance is associated with impairment of leptin transport across the blood-brain barrier (BBB), reduction of the leptin-mediated JAK-STAT signaling, and induction of suppressor of cytokine signaling-3 (SOCS-3) [8]. Among STAT isoforms, STAT3 is proved a major member involving in the LepRb signaling [36]. Thus, we investigated the role of TNF-*α* in changes of LepRb, STAT3, and SOCS-3 protein level in this study. We found that TNF-*α* significantly inhibited LepRb expression and the activation of STAT3, but had no effect on the expression of SOCS3. These results are indicative of the JAK2/STAT3 pathway may be involved in the regulation of TNF-*α* in inhibiting the effects of leptin on *β* cell function.

Transcription factor FOXO1 was reported to modulate the production and secretion of various cytokines and chemokines [47]. Previous studies have demonstrated that FOXO1 involves in insulin resistance-related proinflammatory cytokine production in hepatocytes [21]. In insulin resistance, nuclear FoxO1 expression increases and coactivates with PGC-1*α*, thereby regulating downstream target genes to promote gluconeogenesis [48]. It is reported that insulin resistance does not lead to T2DM unless it is accompanied by pancreatic *β* cell dysfunction, because healthy *β* cells can compensate for insulin resistance by increasing in number and functional output. Despite the FoxO1 central role in the progression of insulin resistance, the effect of FoxO1 on *β* cell function has not been explored in detail and remains somewhat conjectural. Thus, we sought to test the hypothesis that FoxO1 involves in the regulation of TNF-*α* in *β* cell function and to identify the underlying mechanism.

**Figure 8:**
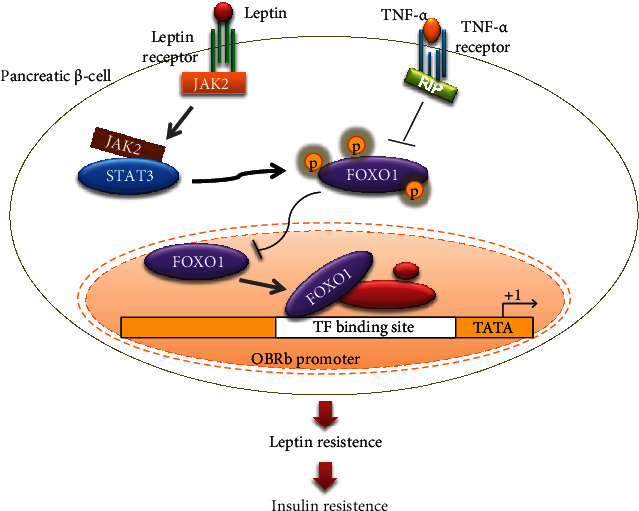
Proposed model for the FOXO1/LepRb pathway to induce *β* cell dysfunction in the T2MD with inflammatory infiltration. Normally, leptin regulates the insulin metabolism of *β* cells through the JAK/STAT3 pathway. In the obesity state, phosphorylated FOXO1 is reduced by inflammatory factor TNF-*α*, and dephosphorylated FOXO1 is translocated into the nucleus to regulate transcription of LepRb gene. FOXO1 transcriptionally reduce LepRb via directly binding to the LepRb promoter as a negative regulator, thereby inhibiting the expression of LepRb gene and reducing the leptin signaling pathway, and ultimately induced leptin resistance and *β* cell dysfunction in T2MD. RIP: receptor-interacting protein.

In the present study, we found that TNF-*α* reduced the phosphorylation level of FOXO1, thus leading to an increase of nucleus FOXO1 in the INS-1E cells. Accumulating evidence has proven that FOXO1 is involved in the insulin signaling pathway as a regulator and can directly bind to a series of target [49]. Thus, we hypothesized that FOXO1 might also interact with LepRb promoter via a transcriptional network. Consistent with the assumptions, we have found that FOXO1 directly binds to LepRb promoters. We have also demonstrated that TNF-*α* reduces LepRb expression, and this effect is significantly reinforced when FOXO1 is overexpressed and attenuated when endogenous FOXO1 is silenced. These data have confirmed that FOXO1 directly binds to the LepRb promoter as a negative regulator, thereby inhibiting the expression of LepRb, and ultimately reduced the leptin signaling pathway. Therefore, we pursued the hypothesis that preventing the transfer of dephosphorylated FOXO1 into the nucleus represents as a means of enhancing leptin receptor signaling to recover leptin pathway which was reduced by inflammatory factors, and a cell signaling mechanism diagram is shown in Figure 8.

In conclusion, our studies based on INS-1E cells in vitro have clarified the molecular mechanism of TNF-*α* aggravating obesity-related *β* cell dysfunction and described a novel function of FOXO1 involved in TNF-*α*-induced LepRb reduction of INS-1E cells. Thus, we conclude that TNF-*α* disrupts the regulation of leptin on the *β* cell GSIS function by inhibiting leptin receptor LepRb and thus producing excessive insulin secretion related to hyperinsulinemia, which is postulated to be a cause of insulin resistance. Our findings may provide a potential therapeutic means for the treatment of obesity-induced hyperinsulinemia and T2DM. This study has potential limitations. We have not elucidated the precise mechanism by interactions between TNF-*α* and the loss of insulin receptor and subsequent insulin resistance. In addition, we confirmed four binding sites of FOXO1 in LepRb promoter, but we have not explored the priority of FOXO1 for these binding sites, and more studies are required to solve these questions.

## Data Availability

All data generated or analyzed during this study are included in this published article.

## Supplementary Materials

Supplementary Materials Primers and reaction conditions of real-time PCR and semiquantitative RT-PCR are shown in Table S1 and Table S2, respectively. Primers used for construction of H-LEPR promoter fusion reporter are shown in Table S3. The complete sequence of LepRb promoter LR3 (-1824/+105 bp) is shown in Figure S1.
