# Supplementary material for: TNF-α Antagonizes the Effect of Leptin on Insulin Secretion through FOXO1-Dependent Transcriptional Suppression of LepRb in INS-1 Cells
Source: Oxid Med Cell Longev. 2022 Feb 14;2022:9142798. doi: 10.1155/2022/9142798 (PMC8860543; doi:10.1155/2022/9142798)
Supplement: Supplementary Materials — Primers and reaction conditions of real-time PCR and semiquantitative RT-PCR are shown in Table S1 and Table S2, respectively. Primers used for construction of H-LEPR promoter fusion reporter are shown in Table S3. The complete sequence of LepRb promoter LR3 (-1824/+105 bp) is shown in Figure S1. [file 9142798.f1.docx]

**Supplementary Materials**

**Table 1S Primer sequence and the reaction conditions for real-time PCR**

| **Genes** | **Primer sequence** (5’→3’) | **Annealing Tm** (°C) | **Cycle** | **Length** (bp) |
| --- | --- | --- | --- | --- |
| Proinsulin  (NM_019129) | (F) cctgcccaggcttttgtca | 60 | 40 | 231 |
|  | (R) ggtgcagcactgatccacaatg |  |  |  |
| LepRb  (NM_012596) | (F) cctcttgtgtcctgctgctcgg | 60 | 40 | 368 |
|  | (R) tccctgggtgctctgagccc |  |  |  |
| SOCS3  (NM_053565) | (F) tggtcacccacagcaagttt | 60 | 40 | 256 |
|  | (R) tgtcgcggataagaaaggtg |  |  |  |
| β-actin  (NM_031144) | (F) gtgggccgccctaggcacca | 60 | 40 | 250 |
|  | (R) cggttggccttagggttcagaggg |  |  |  |

**Table 2S Primer sequence and the reaction conditions for semi-quantitative RT-PCR**

| **Genes** | **Primer sequence** (5’→3’) | **Annealing Tm** (°C) | **Cycle** | **Length** (bp) |
| --- | --- | --- | --- | --- |
| Proinsulin  (NM_019129) | (F) cacctttgtggtcctcacct | 55 | 35 | 168 |
|  | (R) aacctccagtgccaaggtct |  |  |  |
| LepRb  (NM_012596) | (F) ggcacaaggacttaatttcca | 49 | 35 | 200 |
|  | (R) tggaatctggagtggtcaaa |  |  |  |
| SOCS3  (NM_053565) | (F) tggtcacccacagcaagttt | 49 | 30 | 216 |
|  | (R) tgtcgcggataagaaaggtg |  |  |  |
| β-actin  (NM_031144) | (F) agccatgtacgtagccatcc | 58 | 25 | 115 |
|  | (R) aacctccagtgccaaggtct |  |  |  |

**Table 3S Primers used for H-LEPR promoter fusion reporter construction**

| LepRb promoter region LR1 (-3296 / +105 bp):  Forward: 5’- GCACGCGTGAATGGATTTGATGCCCTGT -3’  Reverse: 5’- GCCTCGAGTTTAACGCCCGCCATGTCT -3’ |
| --- |
| LepRb promoter region LR2 (-2376 / +105 bp):  Forward: 5’- GCACGCGTCAGGGCTTGTCTGATACGCAGG -3’  Reverse: 5’- GCCTCGAGTTTAACGCCCGCCATGTCT -3’ |
| LepRb promoter region LR3 (-1824 / +105 bp):  Forward: 5’- GCACGCGTGTGCTTTTCTAGTACCGTGTAC -3’  Reverse: 5’- GCCTCGAGTTTAACGCCCGCCATGTCT -3’ |


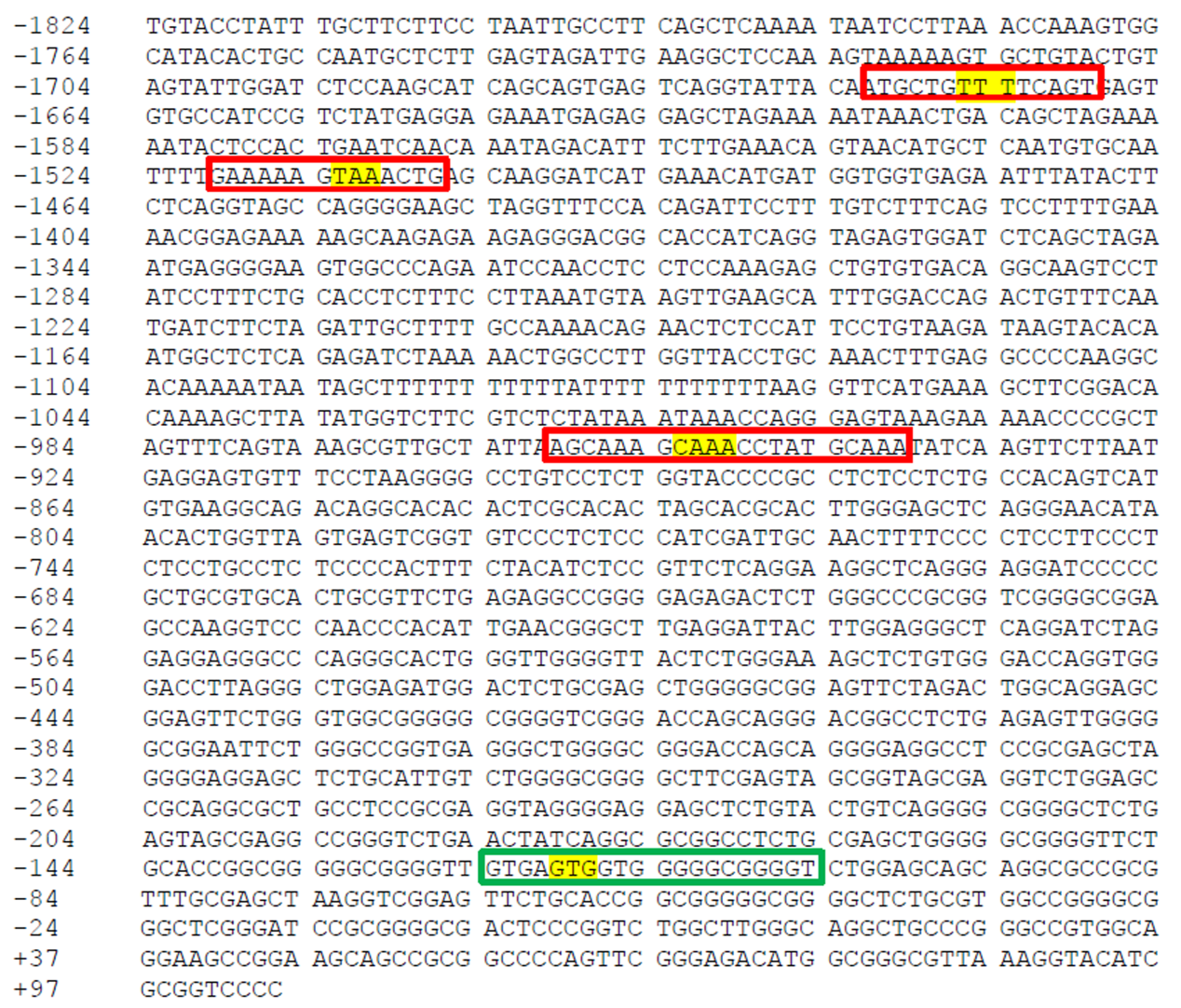


**Figure 1S The complete sequence of LepRb promoter LR3 (-1824/+105bp).** The regions in the red box represent the predicted sense binding site of FOXO1 on LR3, and the region in the green box represent the antisense binding site of FOXO1. The base in the yellow is the mutation sites of these regions.
